# Enhanced carbon-sulfur cycling in the sediments of Arabian Sea oxygen minimum zone center

**DOI:** 10.1038/s41598-018-27002-2

**Published:** 2018-06-06

**Authors:** Svetlana Fernandes, Aninda Mazumdar, Sabyasachi Bhattacharya, Aditya Peketi, Tarunendu Mapder, Rimi Roy, Mary Ann Carvalho, Chayan Roy, P. Mahalakshmi, Rheane Da Silva, P. L. Srinivasa Rao, Suman Kumar Banik, Wriddhiman Ghosh

**Affiliations:** 1 CSIR-National Institute of Oceanography, Dona Paula, Goa, 403004 India; 2 Department of Microbiology, Bose Institute, Kolkata, 700054 West Bengal India; 3 Department of Chemistry, Bose Institute, 93/1 APC Road, Kolkata, 700009 India; 4 Indian Institute of Engineering Science and Technology, Shibpur, Howrah, 711103 West Bengal India; 5 Prima Dona Homes, Dona Paula, Goa, 403004 India; 6 Gujarat Energy Research and Management Institute, Gujarat, 382421 India

## Abstract

Biogeochemistry of oxygen minimum zone (OMZ) sediments, which are characterized by high input of labile organic matter, have crucial bearings on the benthic biota, gas and metal fluxes across the sediment-water interface, and carbon-sulfur cycling. Here we couple pore-fluid chemistry and comprehensive microbial diversity data to reveal the sedimentary carbon-sulfur cycle across a water-depth transect covering the entire thickness of eastern Arabian Sea OMZ, off the west coast of India. Geochemical data show remarkable increase in average total organic carbon content and aerial sulfate reduction rate (J_SO4_^2−^) in the sediments of the OMZ center coupled with shallowing of sulfate methane transition zone and hydrogen sulfide and ammonium build–up. Total bacterial diversity, including those of complex organic matter degraders, fermentative and exoelectrogenic bacteria, and sulfate-reducers (that utilize only simple carbon compounds) were also found to be highest in the same region. The above findings indicate that higher organic carbon sequestration from the water-columns (apparently due to lower benthic consumption, biodegradation and biotransformation) and greater bioavailability of simple organic carbon compounds (apparently produced by fermetative microflora of the sediments) are instrumental in intensifying the carbon-sulfur cycle in the sediments of the OMZ center.

## Introduction

**Figure 1:**
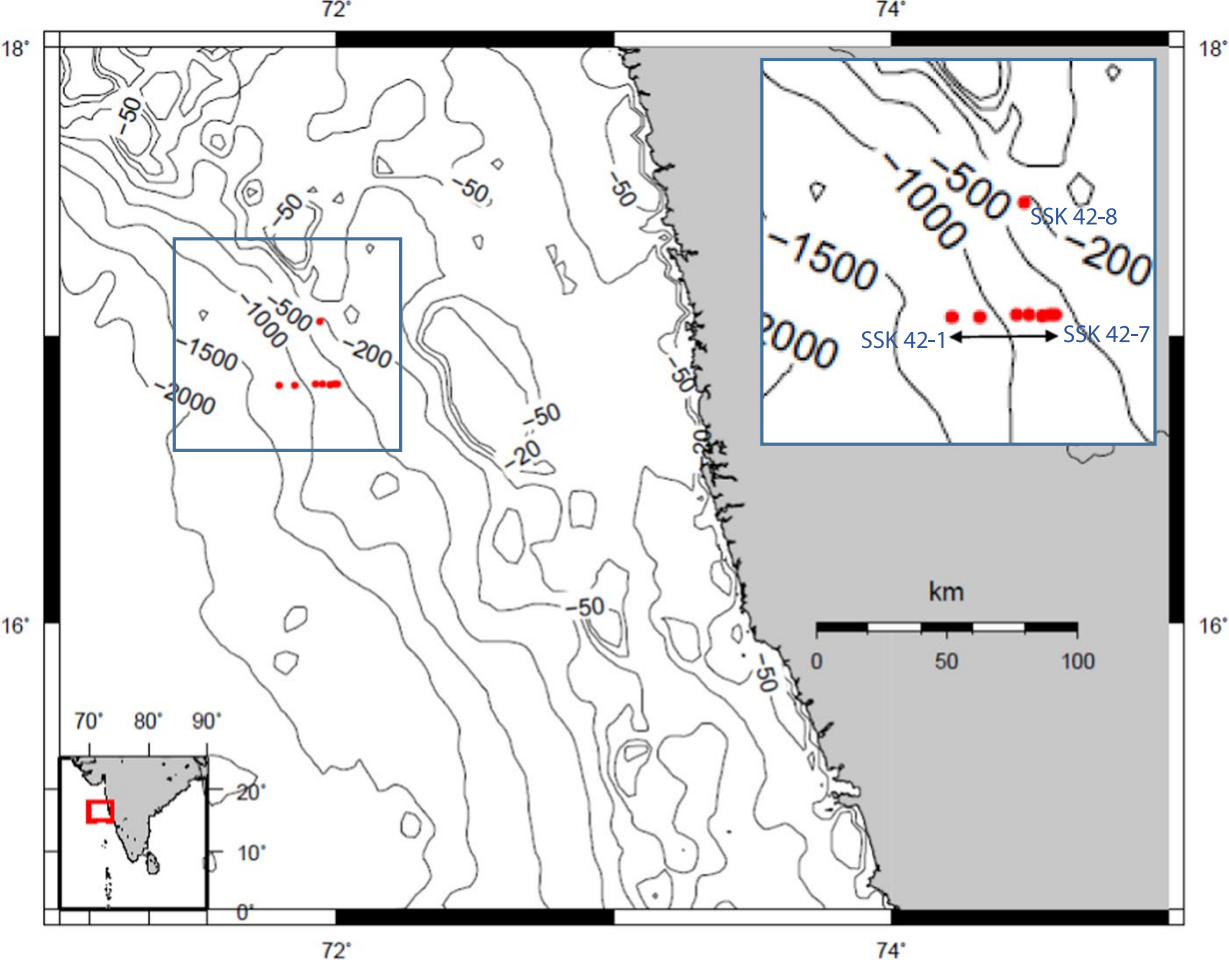
Bathymetry map showing the coring sites (red dots) of this study. The sampling locations are magnified in the inset.

Oxygen minimum zones (OMZs) of the global ocean encompass perennially oxygen-depleted (<20 µM dissolved O_2_) subsurface water masses spanning approximately 100 and 1000 mbsl^1^, and have critical implications for the biodiversity/productivity of the ocean biome^2–4^. The Eastern tropical North Pacific, Eastern tropical South Pacific and Arabian Sea harbor some of the most prominent OMZs defined by secondary nitrite maxima (>0.5 µM NO_2_^−^)^5,6^, arising out of active heterotrophic denitrification^7^ in the water-column. Among the OMZs, the central Arabian Sea contains the thickest (~1.2 km vertical expanse)^8^ and most intensely oxygen-depleted water mass^9^, with the total OMZ area covering approximately 3.2 × 10^6^ km^2^ ^4^. The Arabian Sea OMZ originates from high productivity in the euphotic zone juxtaposed with high respiratory oxygen demand for organic matter degradation^10^ and poor intermediate water ventilation caused by the landlocked geography of the northern portion of the sea^11^. High productivity, in turn, is driven by upwelling of nitrate-rich water mass due to monsoonal current and winter convective mixing^12^. Even though the most intense upwelling occurs along the western boundary of Arabian Sea^13^, the zone of denitrification, remarkably, occurs in the central and eastern regions of the sea^5,8,13,14^. This is potentially attributable to the eastward advection of organic matter from the western side. As the oxygen-deficient water mass impinges upon a large area (1.15 × 10^6^ km^2^)^15^ of the upper continental slope of the Arabian Sea, high primary productivity, lack of oxic respiration and zooplankton grazing^16^ result in organic matter enrichment in the underlying sediments, which in turn become a hotspot of carbon-sulfur sequestration and cycling^17–22^. Enhanced preservation of labile organic matter in anoxic sediments^16^ fuels an intricate network of microbe-mediated anaerobic processes such as organic compounds fermentation, methanogenesis/methanotrophy, sulfate reduction and sulfide oxidation^23–26^. However, compared to the other major OMZs of the world^27–32^ relatively less information is available on the sediment carbon-sulfur biogeochemistry of the Arabian Sea (moreover, most of the latter studies are centered off the Oman and Pakistan margins)^24,33–36^. So far as the overall geomicrobiology of Arabian Sea OMZ sediments are concerned, apart from few recent studies on reflection of climatic oscillation within the microbiome^37^ and microbial characterization coupled with enzymatic diversity^38^, such studies are rare that integrate solid-phase/pore-fluid chemistry with comprehensive microbial diversity data obtained using high-throughput metagenomics. Furthermore, reports of this kind of holistic study of *in situ* carbon-sulfur cycling, involving many discretely located sediment cores from across a transect covering the entire thickness of the OMZ, are practically nil. In this scenario, the present cross-disciplinary study characterizes sedimentary carbon-sulfur chemistry and total bacterial diversity along eight discrete gravity cores (∼3 m long) collected from water-depths spanning 225 and 1275 mbsl, covering the entire thickness of the Eastern Arabian Sea OMZ. These cores collected on-board *RV* Sindhu Sankalp (SSK42), and designated as SSK42/1 through 8, were located across a ∼30 km transect spread across the upper continental slope, off the West coast of India (see Fig. 1 and Supplementary Table 1). Results of this geomicrobiological investigation revealed marked water-depth-dependent spatial variations in (i) depth-integrated sulfate reduction rate; (ii) total bacterial diversity, as well as the diversities of complex organic matter hydrolyzing bacteria, fermentative bacteria and sulfate-reducing bacteria; (iii) organic carbon content; and (iv) shallowing of the depth of sulfate-methane transition zone; and (v) pore-water sulfide buildup. In view of the potential expansion of global OMZs in the recent times, and in the light of the ecologically damaging H_2_S expulsion events reported from sediments off the Peru-Chile and Namibian shelves^39–41^, our findings assume critical oceanographic significance.

## Results and Discussion

### High organic carbon sequestration boosts microbial diversity in sediments underlying the OMZ center

**Figure 2:**
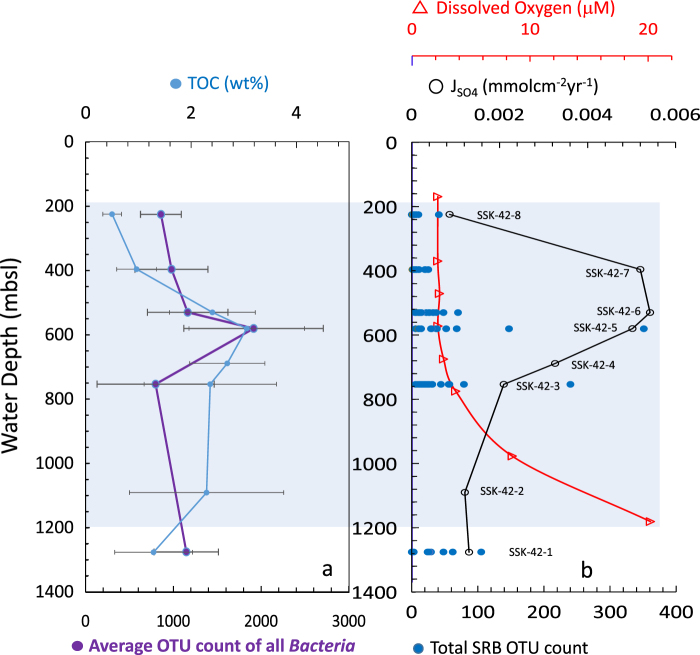
Trends of water-depth dependent variation of key geochemical and microbiological parameters of the Arabian Sea OMZ sediments. (**a**) Variations in the average TOC content of the studied sediment cores (light blue curve), and the average OTU-count of all *Bacteria* (purple curve) in the sediment cores. The error bars in the average TOC plot indicate standard deviation. The range of OTU-count of all *Bacteria* in the relevant sediment cores can be seen from Supplementary Table 9. (**b**) Variations in the bottom-water oxygen concentration (red triangle), sulfate flux J_SO4_ (black circles), and total SRB-OTU-count (blue dots) in the studied sediment cores. The range of SRB-OTU-count in the relevant sediment cores can be seen from Supplementary Table 9. The light blue shaded region represents the thickness of oxygen minimum zone.

Consistency of the (TOC/TN)_molar_ ratios (13.6 ± 3.4) and δ^13^C_TOC_ (−20.6 ± 1‰) values measured across the water-depth transect (Supplementary Fig. 1 and Supplementary Table 2) suggests that marine productivity^42^ is the dominant source of organic matter in the OMZ sediments. Total organic carbon (TOC) content in the investigated cores vary from 0.32 to 5.0 (wt %) (Supplementary Fig. 2b and Supplementary Table 2). Average TOC contents of the cores exhibited marked water-depth dependent variation: cores located close to the upper (SSK42/8; 225 mbsl) and lower (SSK42/1; 1275 mbsl) boundaries of the OMZ have lower average TOC content than those within the OMZ (Fig. 2a). This is attributable to higher consumption and remineralization of organic matter due to relatively higher availability of dissolved oxygen^43,44^ and abundance of benthic fauna^45,46^ near the edges of the OMZ. In contrast, higher TOC content in the sediments underlying the OMZ center (SSK42/5 and 6, located at 580 and 530 mbsl, respectively) could be resultant of greater organic matter flux from the perennially oxygen-depleted water column and diminutive activity of the benthic biota^43^, which not only leaves organic matter largely unutilized but also contributes to the preservation of its biochemical composition^22,47–49^.

**Figure 3:**
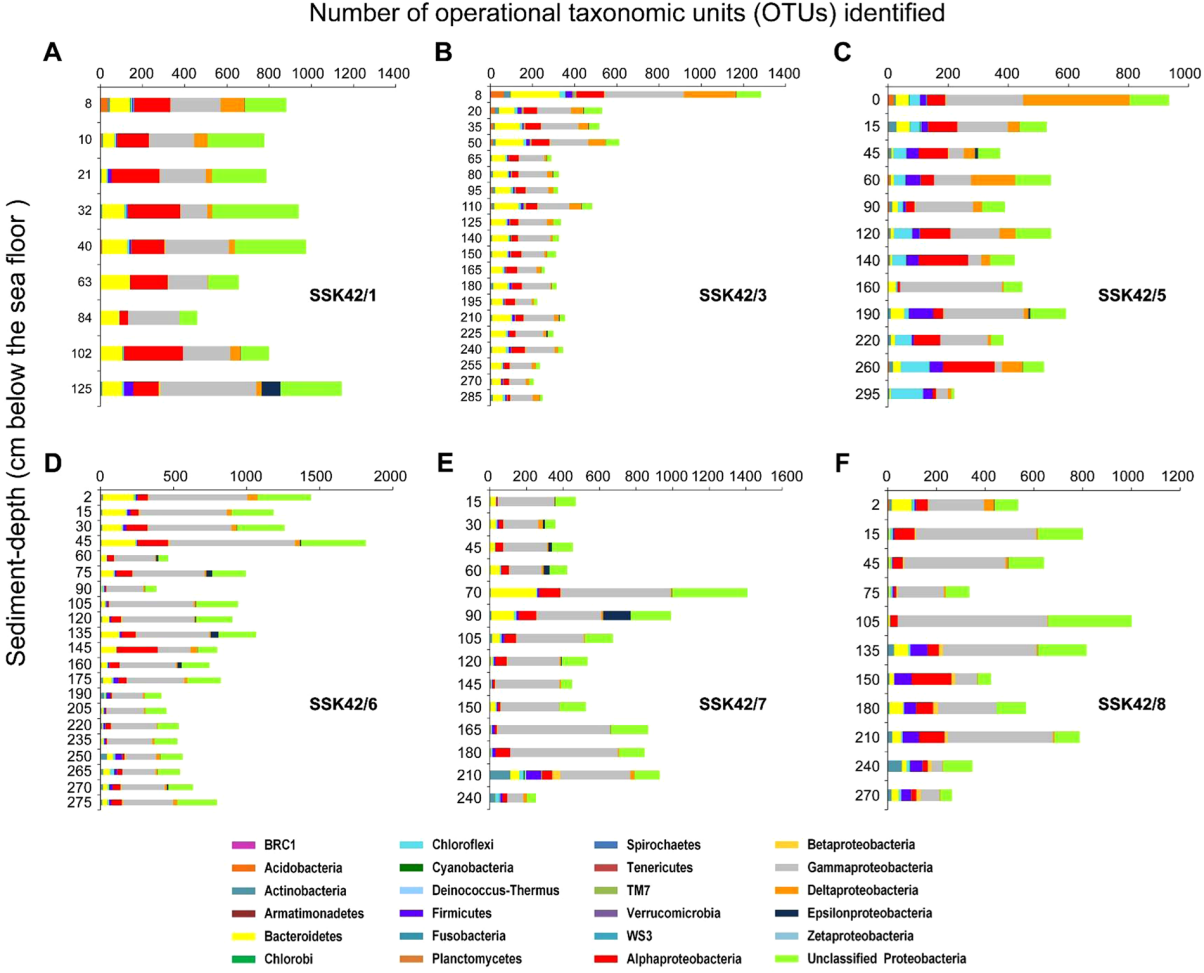
Phylum-/class-level assortment of the taxonomically-classifiable bacterial OTUs identified at the different sediment-datapoints of SSK42/1, 3, 5, 6, 7 and 8. (**A**) SSK42/1; (**B**) SSK42/3; (**C**) SSK42/5; (**D**) SSK42/6; (**E**) SSK42/7; (**F**) SSK42/8. Color-code for the identification of taxa is common for A through F, and is given at the bottom of the figure.

Biodiversity rich, chemoorganoheterotroph dominated microbial communities were found to occur till the bottoms of all SSK42 sediment cores studied for their microbiology (Fig. 3). A maximum of 3374, and a minimum of 332, operational taxonomic units (OTUs, or microbial species-level entities) were identified by PCR-amplification and sequencing (up to a data-throughput level that ensured plateau in the rarefaction curve) of the V3 regions of all bacterial 16S rRNA genes present in the individual metagenomes isolated from the total 87 sediment datapoints studied across SSK42/1, 3, 5, 6, 7 and 8 (Supplementary Tables 3–8). Whereas, *Bacteria*-specific V3 primers generated PCR products of desired size (∼200 bp) from all the metagenomes investigated, *Archaea*–specific primers failed to generate any amplicon from 45 of them, plausibly due to the presence of very low archaeal cell number in the corresponding sediment samples. Notably, deep shotgun sequencing and analysis of whole metagenomes isolated from the sediment datapoints of SSK42/1, 5 and 6 showed that in all the metagenomes for which *Archaea*–specific primers failed to amplify V3 amplicons, archaeal protein-coding and 16S rRNA-encoding reads were ≤0.5% and ≤0.0001% of all annotatable reads, respectively. In contrast, in the same metagenomes, bacterial protein-coding and 16S rRNA-encoding reads were ≥99.5% and 0.1–0.5% of all annotatable reads, respectively. In this way, more than half of the sedimentary communities under investigation had no matching information on their archaeal diversity; the present study therefore focused only on the bacterial component of the communities.

Taxonomic composition of communities were found to vary considerably along the sediment-depths of individual cores, while a number of core-specific variations (for instance, high diversity of *Chloroflexi* in SSK42/5, *Firmicutes* in SSK42/5 and 135-270 cmbsf of SSK42/8) were also apparent across the water-depth transect. However, amidst the variabilities, certain features of global consistency unified microbial community architectures across the sediment cores; these included the presence of diverse *Gammaproteobacteria*, *Alphaproteobacteria* and *Bacteroidetes*, many members of which are chemoorganoheterotrophic and capable of transforming wide range of low to high molecular weight carbon compounds^50–52^. Although the metagenome isolation technique used in the present study did not discriminate between cellular DNA from living and dead microbial cells, or for that matter between cellular DNA and extracellular DNA adhering to the sediment particles, the absence of down-depth decrease in the OTU-count for most of the phyla/classes present suggests that major fractions of the bacterial diversities revealed across the SSK42 cores are viable *in situ*. In the same way as that observed for the average TOC contents of the cores, average OTU count of the sediment cores also exhibited remarkable variation with bottom water oxygen concentration: across the water-depth transect, average OTU count is highest in the sediments of the OMZ center (i.e. for SSK42/5) and relatively lower in the two extremities (Fig. 2a). These findings indicate that dissolved oxygen in the bottom water, and consequentially the TOC content of the sediments, are the key determinants of microbial species richness in the OMZ-underlain sediments.

### Diversity of sulfate-reducing bacteria (SRB) is higher in the sediment horizon of the OMZ center

**Figure 4:**
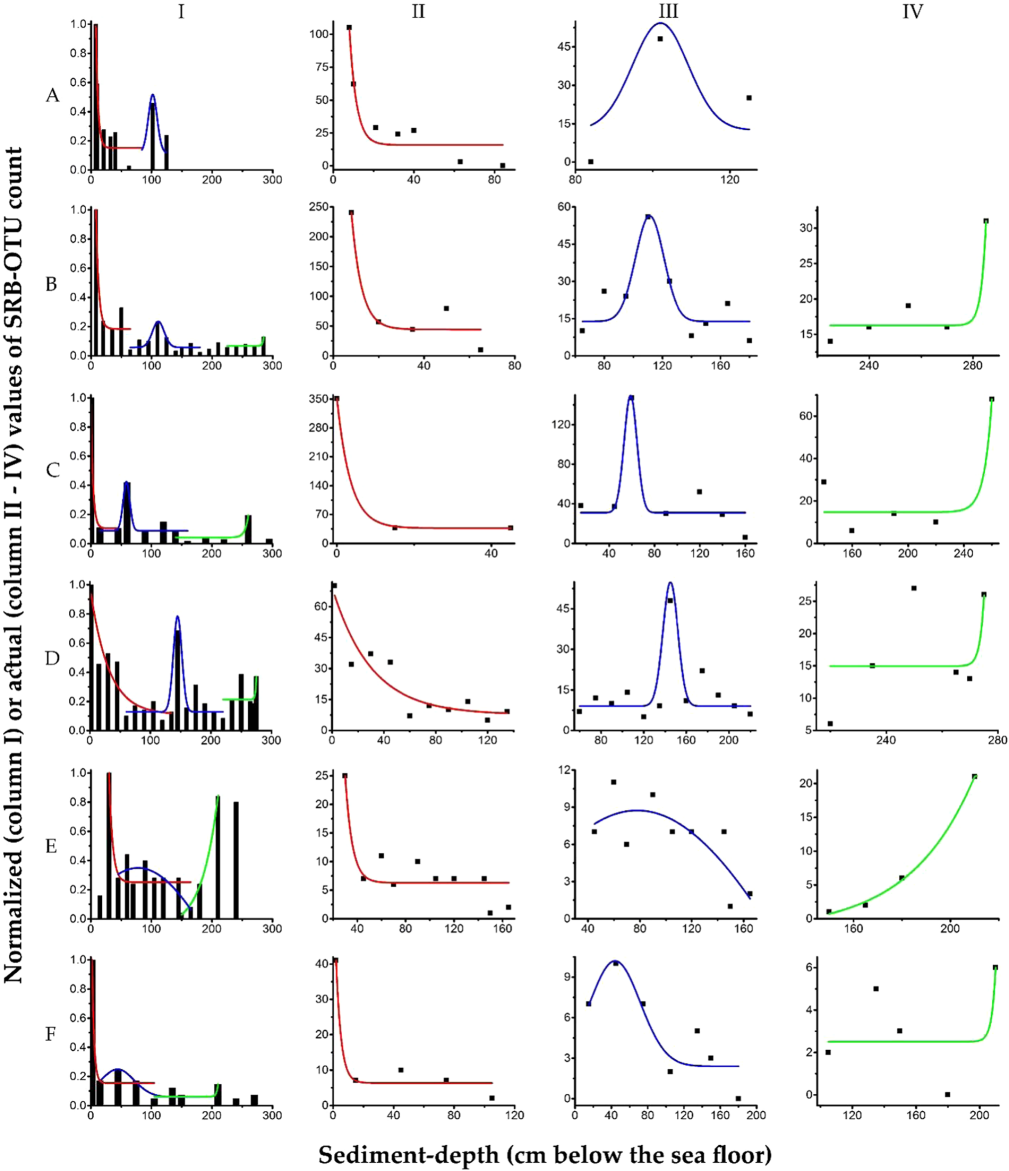
Zones of SRB-OTU-count fluctuation along the sediment cores of SSK42/1 (row **A**), SSK42/3 (row **B**), SSK42/5 (row **C**), SSK42/6 (row **D**), SSK42/7 (row **E**) and SSK42/8 (row **F**), as defined by probability density functions. The histograms shown in the six panels of column I were derived based on the normalized values of the SRB-OTU-counts obtained at the individual sediment-depths of the six coring-stations. The differently-colored lines represent the probability density functions fitting to the different zones of SRB-OTU-count fluctuation. Expanse of the different zones along the sediment-depths of the six coring-station have been shown separately in columns II through IV, where all panels under Column II depict Zone 1 (red lines, which follow exponentially decaying distribution); all panels under column II depict Zone 2 (blue lines, which follow Gaussian distribution; and all panels under column IV depict Zone 3 (green lines, which follow exponential growth).

Almost every sediment sample explored for microbiology across SSK42/1, 3, 5, 6, 7 and 8 were found to encompass at least one OTU ascribable to known sulfate-reducing bacterial taxa (Supplementary Table 9). In this context it is noteworthy that the actual SRB diversity may well be higher for some of the sample sites because the present analyses were based exclusively on bacterial diversity data (see the Methods section “Species-richness estimation for sulfate-reducing bacteria” below) and the sulfate-reducing genera such as *Desulfurococcus*, *Desulfurolobus* (phylum *Crenarchaeota*), and *Archaeoglobus* (phylum *Euryarchaeota*) of the domain *Archaea* may also be present. In all the sediment cores, maximum SRB-OTUs were identified within 30 cmbsf; subsequently, however, multiple phases of rise and fall in SRB diversity were detected down the sediment-depths (Fig. 4; Table 1). Trends of variation in SRB-OTU distribution along the sediment-depth of each core were analyzed by determining the probability density function for every individual zone of fluctuation. In doing so, a common pattern of SRB diversity distribution along the surface-sediment to core bottom trajectory was revealed for the entire region. This involved (i) an upper zone of exponential decay, (ii) a middle zone encompassing a Gaussian distribution, and (iii) a lower zone of exponential rise, in SRB-OTU count (Fig. 4), defined by the probability density functions given in equations (1), (2) and (3), respectively. In these equations, the constants *A*_1_, *t*_1_, *w*, and *x*_*c*_ were computed simultaneously from the iterative fitting of the functions with the OTU-count data (to optimize the probability density functions they were sampled for up to 4000 iterations till the χ^2^ values reached their minima).

1 $${\rm{P}}({\rm{X}})={A}_{1}{e}^{-\frac{x}{{t}_{1}}}$$

2 $${\rm{P}}({\rm{X}})=\frac{A}{w\sqrt{\pi /2}}{e}^{-2\frac{{(x-{x}_{c})}^{2}}{{w}^{2}}}$$

3 $${\rm{P}}({\rm{X}})={A}_{1}{e}^{\frac{x}{{t}_{1}}}$$

**Table 1:** Segmentation^a^ and characterization^b^ of the three zones^c^ of SRB-OTU distribution along the sediment-depths of the six SSK42 cores.

Name of the coring-station	Zone 1 ${\boldsymbol{(}}{\boldsymbol{y}}{\boldsymbol{=}}{{\bf{y}}}_{{\bf{0}}}{\boldsymbol{+}}{{\boldsymbol{A}}}_{{\bf{1}}}{{\boldsymbol{e}}}^{{\boldsymbol{-}}\frac{{\boldsymbol{x}}}{{{\boldsymbol{t}}}_{{\bf{1}}}}}{\boldsymbol{)}}$	Zone 2 ${\boldsymbol{(}}{\boldsymbol{y}}{\boldsymbol{=}}{{\bf{y}}}_{{\bf{0}}}{\boldsymbol{+}}\frac{{\boldsymbol{A}}}{{\boldsymbol{w}}\sqrt{{\boldsymbol{\pi }}{\boldsymbol{/}}{\bf{2}}}}{{\boldsymbol{e}}}^{{\boldsymbol{-}}{\bf{2}}\frac{{{\boldsymbol{(}}{\boldsymbol{x}}{\boldsymbol{-}}{{\boldsymbol{x}}}_{{\boldsymbol{c}}}{\boldsymbol{)}}}^{{\bf{2}}}}{{{\boldsymbol{w}}}^{{\bf{2}}}}}{\boldsymbol{)}}$	Zone 3 ${\boldsymbol{(}}{\boldsymbol{y}}{\boldsymbol{=}}{{\bf{y}}}_{{\bf{0}}}{\boldsymbol{+}}{{\boldsymbol{A}}}_{{\bf{1}}}{{\boldsymbol{e}}}^{\frac{{\boldsymbol{x}}}{{{\boldsymbol{t}}}_{{\bf{1}}}}}{\boldsymbol{)}}$
SSK42/1	D = 0–84 cm χ^2^ = 185.031	D = 84–125 cm χ^2^ = 0.134	—
SSK42/3	D = 8–65 cm χ^2^ = 1190.057	D = 65–180 cm χ^2^ = 62.253	D = 180–285 cm χ^2^ = 6.376
SSK42/5	D = 0–45 cm χ^2^ = 0.624	D = 15–160 cm χ^2^ = 373.333	D = 140–260 cm χ^2^ = 151.524
SSK42/6	D = 2–135 cm χ^2^ = 69.652	D = 60–220 cm χ^2^ = 73.105	D = 220–275 cm χ^2^ = 77.225
SSK42/7	D = 30–165 cm χ^2^ = 11.919	D = 45–165 cm χ^2^ = 6.355	D = 150–240 cm χ^2^ = 0.447
SSK42/8	D = 2–105 cm χ^2^ = 16.333	D = 15–180 cm χ^2^ = 4.704	D = 105–210 cm χ^2^ = 6.001

^a^Expanse of a given zone over a sediment core (i.e. the range of sediment-depth covered by the zone) is denoted by D.

^b^The minimal χ^2^ value that optimized the distribution function of a particular zone in a particular core segment is given next to information on D.

^b^The OTU-distribution function that characterizes a particular zone is shown in parenthesis below the zone name.

Notwithstanding the down-depth fluctuations revealed for the total OTU-count of SRB, genus-level classification of the SRB OTUs showed that members of the deltaproteobacterial orders *Desulfobacterales* and *Desulfovibrionales* are present in all the sediment communities explored for their microbiology. Furthermore, overall SRB diversity for individual sediment cores, across the sampling transect, was found to increase from SSK42/1 through SSK42/5, and then decline SSK42/6 onward (Fig. 2b). The mean, median, maximum, as well as minimum, SRB-OTU-count for a sediment core (as determined from the data presented in Supplementary Table 9) were all found to be highest for SSK42/5.

### Microbial sulfate reduction intensifies in the sediments underlying the OMZ center

**Figure 5:**
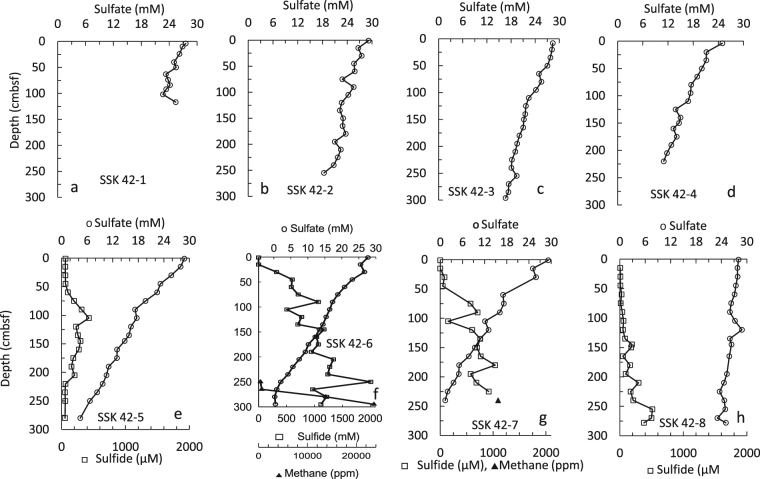
Concentrations of pore-water SO_4_^2−^ (hollow circle), ΣHS^−^ (hollow square) and CH_4_ (filled triangle) in sediment sub-samples in cores SSK42/1-8. The sulfate-methane transition zone (SMTZ) in SSK42-6 is within 250 to 275 cmbsf. The maximum hydrogen sulfide concentration is observed within the SMTZ.

Consumption of pore-water sulfate via organoclastic sulfate reduction (OSR) and anaerobic oxidation of methane (AOM), together with fermentation and methanogenesis, are the major drivers of carbon-sulfur cycle in anoxic sediments^53,54^. In all the SSK42 cores, pore-water sulfate concentration was found to decrease linearly with sediment-depth (Fig. 5; Supplementary Table 2) with the SO_4_^2−^ concentration gradients ranging between 0.015 (for SSK42/8) and 0.11 (for SSK42/7) mM cm^−1^. In this context it is noteworthy that in the gravity cores of the present study, potential loss of core-top sediment layers (that have highest sulfate reduction rates) is unlikely to have any major influence on the sulfate concentration gradients because over the top few centimeters of any sediment package there is virtually no decrease in SO_4_^2−^ concentration due to the short diffusion distance involved^31,54^. Sediment-depth-integrated sulfate reduction rates (J_SO4_^2−^) calculated for the individual cores from their sulfate concentration profiles were found to range between 0.0008 (for SSK42/8) and 0.0113 (for SSK42/7) mmol cm^−2^ yr^−1^ (Fig. 2b). These values represent net sulfate reduction rates that are generally lower than the radiotracer based (^35^S_SO4_^2−^_/HS_^−^) gross sulfate reduction rates^27^, which, unlike the net rates, incorporate the high sulfate reduction rates of the core-tops. Potential underestimations relative to the expected gross rates notwithstanding, the present J_SO4_^2−^ values are well within the range of net sulfate reduction rates reported for sediments underlying the eastern Arabian Sea OMZ, and are also comparable with those obtained for other prominent OMZ sediments^20^. Notably, the J_SO4_^2−^ values calculated along the present water-depth transect reach their maxima within the center of the OMZ (Fig. 2b) and decrease towards the boundaries of its vertical expanse due to the relatively higher oxygen enrichment at the sediment water interface, a phenomenon that has parallels reported from *in vitro* studies on SRB^55,56^. On the other hand, the enhanced sulfate reduction rate observed in the sediments of the OMZ center could be attributable to potentially greater availability of labile organic matter supporting OSR and methanogenesis^57^. While the abundance of labile organic matter (simple short chain fatty acids, such as acetate, lactate, succinate and formate) and H_2_ in sediments are known to determine the extent of OSR, methanogenesis^58,59^ and consequently the depth of sulfate-methane transition zones (SMTZs), it is noteworthy that the frequencies of OTUs affiliated to fermentative^60^ and exoelectrogenic^61^ bacteria, which are potent sources of *in situ* lactate, acetate, CO_2_ and/or H_2_, are also highest along the sediment-depth of SSK42/6, as compared to the other cores (Supplementary Tables 10–15). Additional factors such as sedimentation rate may also be responsible for the spatial variation in sulfate reduction rate; for instance, it is known that slower sedimentation rate may enhance the oxygen exposure time of organic matter^62,63^ at the sediment water interface and in doing to preserve the lability of organic matter crucial for sulfate reduction^64^.

**Figure 6:**
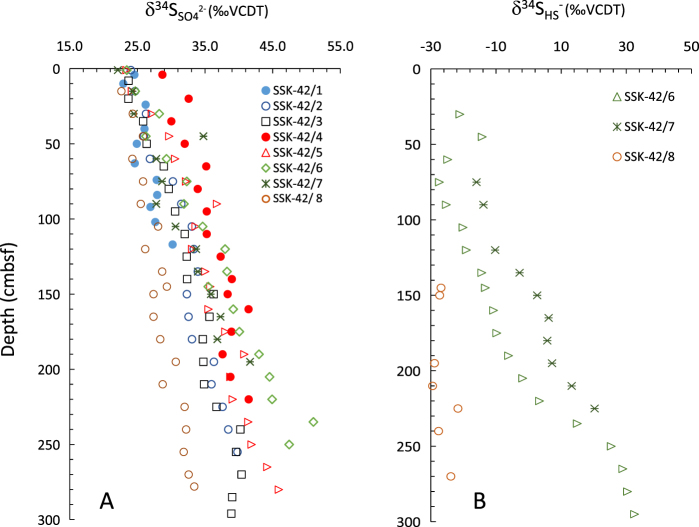
δ^34^S profiles of dissolved sulfate (SO_4_^2−^) and hydrogen sulfide (ΣHS^−^) in SSK42 pore water samples. δ^34^S_SO4_ and δ^34^${{\rm{S}}}_{{{\rm{H}}{\rm{S}}}^{-}}$ profiles show enrichment trends with depth.

Coupled influence of organic matter remineralization and OSR on the SO_4_^2−^ concentration gradients of the SSK42 cores is evident from the down-depth decrease in TOC and SO_4_^2−^, and increase in dissolved NH_4_^+^ and ΣHS^−^ concentrations^65^ (Fig. 5, Supplementary Fig. 2a, Supplementary Table 2). Across the eight cores studied, dissolved NH_4_^+^ ranged between 59 and 2214 µM, whereas ΣHS^−^ was detectable only along SSK42/5, 6, 7 and 8. Across these sediment cores, dissolved ΣHS^−^ concentrations were found to vary between non-detectable and 2010 µM. Down-depth variations in the pore-water SO_4_^2−^ and ΣHS^−^ concentrations are also accompanied by corresponding shifts in sulfur isotope ratios of SO_4_^2−^ and ΣHS^−^. In SSK42/1 through 8, the core-top δ^34^S_SO4_ varies between 22.1 and 28.7‰ VCDT while the down-depth profiles of δ^34^S_SO4_ show enrichment trends for all the eight cores (Fig. 6A). The δ^34^S_SO4_ maxima for the individual sediment cores were found to vary between 30.2 and 51‰ VCDT. δ^34^S_HS_^−^ values recorded along SSK42/6, 7 and 8 vary between −22.4 and +32.5‰ VCDT and exhibit down-depth ^34^S enrichment, as observed for the source SO_4_^2−^ (Fig. 6B). Whereas in SSK42/8, δ^34^S_HS_^−^ ranges from −29.4 to −21.4‰ VCDT with no definite enrichment trend. The down-depth enrichment trend of dissolved pore-water sulfate is attributed to the OSR by obligate anaerobes which preferentially utilize the lighter sulfur isotope ^32^S, leaving pore fluids progressively enriched in ^34^S^66^. In sediment cores studied for sulfur isotopic fractionation (i.e. SSK42/6, 7 and 8), Δ_SO4_^2−^_−HS_^−^ values range between 22 and 59.8 (Avg: 46.7 ± 10.4‰ VCDT) and are well within the range of S-fractionations reported for OSR, with or without disproportionation of intermediate sulfur species (S°, S_2_O_3_^2−^)^67–70^. Notably, genus-level classification of the OTUs identified in the SSK42 cores showed the SRB diversities across the sample-sites were dominated by *Desulfovibrio* species that are known to be capable of sulfur isotopic fractionation up to 46‰^71^. Other sulfate-reducers prevalent in the SSK42 cores include *Desulfococcus*, *Desulfobacteria* and *Desulfotalea spp*., which can render S-isotope fractionation between 16.1 and 32.7‰ VCDT^72^. In this context it is noteworthy that OTUs belonging to the anaerobic/facultatively anaerobic genera *Arcobacter*, *Paracoccus*, *Sulfurimonas*, *Thiohalomonas* and *Thiohalophilus* that are capable of chemolithotrophically-oxidizing reduced sulfur species including sulfide^73^, were detected intermittently along the sediment-depth of every coring station (Supplementary Tables 10–15). However, more information is required to ascertain whether they actually render oxidation of H_2_S (or other intermediate sulfur species) *in situ*, and thereby influence net S-isotope fractionation in the pore-waters.

**Figure 7:**
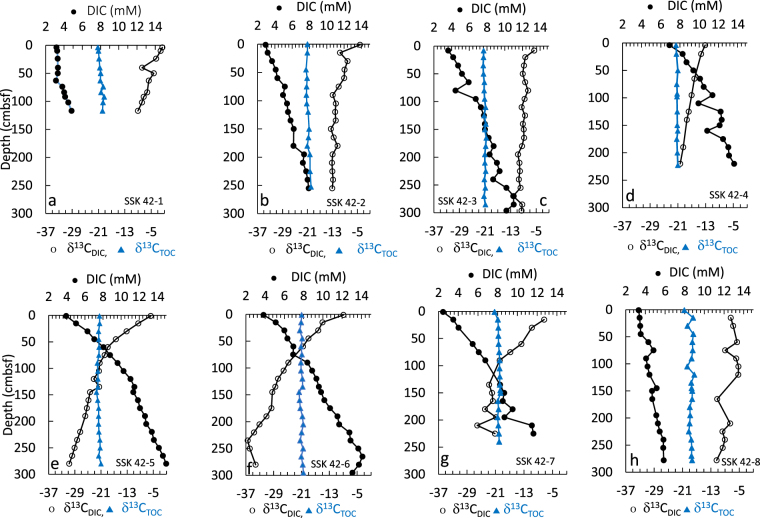
Concentrations of pore-water DIC (filled black circle), and stable carbon isotopic ratios (δ^13^C) of DIC (hollow black cicle) and TOC (filled blue triangle), down the sediment-depths of SSK42/1-8.

AOM is known to play a major role in shaping the sulfate concentration profile of sediments^54,58^. AOM, mediated by a syntrophic consortium of anaerobic methanotrophic archaea (ANME) and SRBs^74,75^, takes place typically in the SMTZs of sediments, and results in the *in situ* enrichment of HCO_3_^−^ and ΣHS^−^. The carbon isotope ratio of dissolved inorganic carbon (DIC) in organic-matter–rich, anoxic marine sediments depends on the mixing of DIC produced via OSR and AOM pathways^53,76^. δ^13^C of DIC produced via OSR approaches the δ^13^C of the *in situ* TOC, whereas δ^13^C of AOM derived DIC is generally much lower than that of the *in situ* TOC. In the SSK42 cores, DIC concentrations increase with depth (DIC varies between 2.4 and 14.9 mM, across the eight cores studied) (Fig. 7). It is noteworthy that below 60–90 cmbsf of SSK42/5, 6 and 7 (the cores underlying the OMZ center), δ^13^C_DIC_ gets significantly depleted (−22 to −36.4‰ VPDB) in comparison to δ^13^C_TOC_ (Fig. 7), thereby indicating strong upward diffusion of ^12^C-enriched DIC produced via AOM at the SMTZ^77,78^. On the other hand, δ^13^C_DIC_ remains much higher than δ^13^C_TOC_ in the other sediment cores investigated, thereby suggesting the deeper occurrence of SMTZs in those areas. Shallow depths (250–275 cmbsf) of SMTZs within the OMZ center (SK42/6 and 7) corroborate the intensification of AOM within this territory.

Carbon isotopic composition of the methane detected in SSK42/6 and 7 (δ^13^C values between −87.8 and −62.2‰ VCDT; Supplementary Table 2) is within the range of δ^13^C values recorded previously for microbial methane^79^. Furthermore, deep shotgun sequencing and analysis of whole metagenomes isolated from the sediment datapoints of SSK42/6 showed that the predominant methanogen in these sediments is *Methanosarcina* (S. Bhattacharya *et al*., Unpublished), which produces methane having −108 to −76‰ δ^13^C^80,81^.

### Bioavailable organic carbon is the potential driver of intense biogeochemical activity in the sediments underlying the OMZ center

Characteristic features such as (i) enhanced TOC content (ii) tandem increase in J_SO4_^2-^ and SRB-diversity, (iii) shallowing of SMTZ, and (iv) buildup of high ΣHS^−^, are all reflective of heightened carbon-sulfur cycling in the sediments underlying the center of the eastern Arabian Sea OMZ. In addition, increase in total bacterial diversity within this region implicates high organic matter input as the potential trigger for the intensification of the entire biogeochemical cascade. Collectively, these features suggest that high as well as low molecular weight carbon compounds, utilizable by complex organic matter degraders and sulfate-reducers respectively, are abundant under the OMZ center. Greater preservation of labile organic matter within the OMZ center has also been insinuated in previous studies that measured chlorophyll degradation products in OMZ sediments across the eastern Arabian Sea^82^. In this context it is further noteworthy that although TOC and SRB diversity reach their maxima within the zone of high J_SO4_, water-depth dependent variation profiles of the first two parameters do not exactly overlap with that of the third (Fig. 2b). We therefore suggest that the potential nature of the bioavailable organic-compounds is a key determinant of *in situ* sulfate reduction rate, over and above TOC content and SRB diversity. Corroborating this conclusion, J_SO4_^2−^ drops sharply beyond the OMZ center, concomitant to the rise in bottom-water oxygen, thereby pointing towards the progressive reduction in the burial of bioavailable organic matter (from the water columns) owing to oxygen influenced increase in benthic consumption, biodegradation and biotransformation of organic matter^83^.

In a nutshell, our study identifies the sediments impinged by the hypoxic waters of the OMZ center as major sinks of marine organic matter sequestration, and hotspots of carbon-sulfur cycling. The present findings hold implications for the predictive modeling of potential loci of hydrogen sulfide and methane expulsions, which can damage the pelagic and benthic biota severely. Solid phase analyses of these sediments might offer more insights into the past variations in AOM intensity, SMTZ depth, and overall expanse of the OMZ^84,85^.

## Methods

### Coring and CTD profiling

Eight gravity cores were collected onboard RV Sindhu Sankalp (SSK-42) in December 2012 across the upper continental slope along the western continental margin of India (Supplementary Table 1 and Supplementary Fig. 1). Barring core SSK42/1 (water-depth: 1275 mbsl), all other cores are located within the perennial oxygen minimum zone. Sea water-depth, temperature, salinity and oxygen concentrations were measured using a SBE 911plus CTD profiler (Sea Bird Electronics, Bellevue, Washington, USA) in the vicinity of the coring locations. (Supplementary Table 1 and Supplementary Fig. 1).

### Sampling for geochemistry, and porosity measurement

Cores were sub-sampled on-board for hydrocarbon gas analysis and pore-water extraction. For hydrocarbon gas analyses, sediment was extracted by inserting 10 ml cut syringe deep into the core at an interval of 15 cm. Sediment was transferred to 28 ml glass vials containing 10 ml of sodium azide to arrest microbial activity. The vials were flushed with helium, crimped and stored at 4 °C until further analysis.

For extraction of pore-water, 50 ml cut syringes were inserted deep into the core, at an interval of 15 cm. Sediment was transferred from the syringe into 50 ml centrifuge tubes (Tarsons Products Private Limited, Kolkata, India) under a stream of nitrogen to avoid oxidation of dissolved sulfide. The tubes were centrifuged at 4700 g and 4 °C for 15 minutes in a C30 cryocentrifuge (REMI Laboratory Instruments, Mumbai, India). The supernatant was filtered through 0.22 µm Whatman syringe filter and the aliquots were transferred into glass vials (Supelco Inc, Bellefonte, PA, USA) for measurement of concentrations and isotope ratios of various constituents. Dissolved ΣHS^−^ was precipitated as CdS by the addition of CdNO_3_. The vials were crimped immediately following nitrogen flushing and stored at 4 °C until further analysis. Sulfate concentration was measured in the ΣHS^−^ free supernatant pore-water.

Sub-sampling of cores for solid phase analysis was carried out as 1 cm slabs. The sub samples were stored at 4 °C until further analysis. For porosity measurements, measured volume of sediment was dried at 105 °C. Moisture content in the sediment was calculated from the difference in wet and dry weight of sediments. Porosity was calculated as: (volume of sediment pore-water/wet sediment volume weight) × 100.

### Measurement of concentrations and isotope ratios

Pore-water sulfate concentrations were measured by a Basic IC plus 883 ion chromatograph (Metrohm AG, Herisau, Switzerland) equipped with a suppressed conductivity detector (Metrohm, IC detector 1.850.9010) and a MetrosepASupp 5 (150/4.0) anion exchange column (Metrohm AG, Herisau, Switzerland). A mixed solution of 1 mM NaHCO_3_ and 3.2 mM Na_2_CO_3_ was used as the eluent and 0.2 N H_2_SO_4_ as the regeneration fluid. Pore-water samples were diluted 1000-fold with de-ionized water (Siemens, <0.06 µS) prior to analysis. A 10 ppm Fluka mixed anion standard was used to prepare the calibration curve for quantification. The sample reproducibility was ±0.2 ppm. Dissolved sulfide concentrations were measured from the CdS precipitate following methylene blue complexation method. The absorbance of methylene blue complex was measured at 670 nm using a spectrophotometer (Spectrascan UV 2700, Chemito Instruments, Mumbai, India). Carbon coulometer (CM5130) was used to determine the concentration of dissolved inorganic carbon. Ammonia was determined using the indophenol blue method. Absorbances were measured at 640 nm using the spectrophotometer mentioned above.

Concentration and carbon isotopic composition of hydrocarbon gases present in the head space samples was determined using a continuous flow isotope ratio mass spectrometer (Delta V Plus, Thermo Fisher Scientific, Germany) coupled with a gas chromatograph (Thermo Fisher Scientific, Germany). Peak area calibration curve was prepared using standard hydrocarbon gas mixtures for concentration measurement. Carbon isotope ratios are reported in standard format relative to the Vienna Peedee Belemnite (VPDB) standard. The external precision calculated for δ^13^C is typically 0.07–0.09‰ (VPDB). δ^13^C of DIC was analyzed on a isotope-ratio mass spectrometer (Thermo Finnigan Delta^Plus^ XP) coupled to a Thermo Finnigan Gasbench II at the Stable Isotope Geochemistry Laboratory, University of Kentucky, USA. Pore-water sulfate was precipitated as BaSO_4_ by the addition of 1 ml of 1 M BaCl_2_ solution following acidification. Dissolved sulfide precipitated as CdS was filtered through a 0.22 µm nitrocellulose filter paper. The precipitate was converted to silver sulfide (Ag_2_S) by the addition of silver nitrate (AgNO_3_) solution. The dried and homogenized BaSO_4_ and Ag_2_S precipitates were mixed with V_2_O_5_ and flash combusted at 1150 °C in an elemental analyzer (EA1112, Thermo Fisher Scientific, Germany) and subsequently analysed for stable sulfur isotope ratios using a continuous flow isotope ratio mass spectrometer (Thermo Delta V plus, Thermo Fisher Scientific, Germany). All results are reported in standard delta notation (δ^34^S) as per mil deviations from the VCDT (Vienna Canyon Diablo Troilite). IAEA standards S-1, S-2, S0-5 and S0-6 were used for instrument calibration. Sample reproducibility of ±0.3‰ is reported here.

TIC content of standard reference material (Ultrapure CaCO_3_ from Sigma-Aldrich) was 12.0 ± 0.25%. Total carbon (TC) and nitrogen (TN) content of freeze dried and desalinated sediment samples were measured by EA1112 elemental analyzer (Thermo Fisher Scientific, Germany). Total organic Carbon (TOC) content was calculated by subtracting TIC from TC. 2,4-DNP was used as a calibration standard for TC. Reproducibility for TC in NIST-SRM1944 sediment standard was found to be 4.4 ± 0.2% and TN in MAG-1 sediment standard was found to be 0.24 ± 0.03%. δ^13^C_TOC_ was measured on decarbonated sediments using a Delta-V-plus isotope ratio mass spectrometer coupled with an EA1112 elemental analyzer (Thermo Fisher Scientific, Germany). The external standard reproducibility calculated for δ^13^C_TOC_ using cellulose is typically −24.7 ± 0.1‰ (VPDB).

### Depth-integrated Sulfate flux calculations

Diffusive sulfate flux (J_SO4_) was calculated from concentration profiles using Fick’s first law assuming steady state conditions.

4 $${{\rm{J}}}_{{\rm{SO}}4}={\rm{\phi }}\text{Ds}\,(\text{dC}/\text{dX})$$

Where J, C and φ represent the depth-integrated sulfate reduction rate (mmol cm^−2^ yr^−1^), sulfate concentration (mM) in the pore-water and average sediment porosity respectively. dC/dX is the sulfate concentration gradient and Ds (cm^2^ s^−1^) is the molecular diffusivity corrected for tortuosity. Ds is calculated by the formula:

5 $${\rm{Ds}}={\rm{Do}}/[1+{\rm{n}}(1-{\rm{\phi }})]$$

where n = 3 for clays and silt^86^. Do is the sulfate diffusivity in the absence of particles. Since Do varies with temperature (water-depth), it was calculated for the varying bottom water temperatures using the empirical relation based on previous data^87^.

### Sampling for studies of *in situ* microbial ecology, and isolation of metagenomic DNA from the sediment samples

For microbiological investigations, sediment samples were collected onboard, aseptically from various sediment-depths of SSK42/1, 3, 5, 6, 7 and 8, immediately after cutting and removal of longitudinal halves of the PVC core-liners. Identities of the 87 sediment-datapoints investigated for microbiology across the six coring stations are given in Supplementary Tables 3–8. As mentioned above, the top one-cm of the exposed surface was first removed so as to avoid atmospheric and sea water contamination; sampling was carried out strictly under nitrogen flow. In order to sample a given sediment-depth from a given core (i.e. a particular sediment-datapoint), an approximately 5-mm-thick sediment-slice (spanning equally on either side of the core-height marking) was scooped out with sterile scalpels and put into a sterile polypropylene bottle. The head-space of every sample-containing bottle was flushed with nitrogen gas, following which it was sealed with Parafilm (Neenah, WI, USA) and immediately placed under refrigeration: samples meant for metagenome analysis and culture-based study (the latter is not a part of this paper) were stored at −20 °C and 4 °C, respectively. From the laboratory onboard SSK-42, *en route* to the laboratory at Bose Institute (Kolkata) these sample-preservation temperatures were maintained all-through. Upon reaching the laboratory, extraction of total community DNA (metagenome) from every sediment sample was completed within a week. Metagenomes were isolated using PowerSoil DNA Isolation Kit (MoBio, Carlsbad, CA, USA), as per manufacturer’s protocol.

### Taxonomic diversity estimation in the sedimentary microbial communities

Taxonomic composition of the microbial communities occurring at various sediment-depths of SSK42/1, 3, 5, 6, 7 and 8 was determined by high-throughput sequencing and analyses of the V3 regions of all 16S rRNA genes present in the respective metagenomes. Following methods described earlier^88,89^, V3 regions of all *Bacteria*- or *Archaea*-specific 16S rRNA genes that were present in the metagenome of a given community were PCR-amplified separately using domain-specific oligonucleotide primer-pairs and sequenced by the Ion Torrent Personal Genome Machine (Ion PGM; Thermo Fisher Scientific, Waltham, Massachusetts, USA) up to such a data-throughput level that ensured plateau in the rarefaction curve. Subsequently, all the V3 reads present in a given sequence dataset were clustered (at 97% sequence similarity level) into operational taxonomic units (OTUs) or potential microbial species-level entities (see below); taxonomic classification of these OTUs revealed the total diversity of the community.

### Amplification of 16S rRNA gene fragments and sequencing by Ion PGM

Amplification of 16S rRNA gene fragments and sequencing by Ion PGM was carried out using the Fusion Primer protocol, as described previously^88,89^. V3 regions of all potential bacterial or archaeal 16S rRNA genes present in the studied sample were amplified by polymerase chain reaction (PCR) using domain-specific oligonucleotide primers. In order to enable tandem sequencing of multiple samples on a single chip each sample DNA was subjected to PCR using a 16S forward primer prefixed with an Ion Torrent adapter and a unique sample-specific barcode or multiplex identifier in the following order in the 5′ to 3′ direction: (i) a 26-mer A-linker followed by a 4-mer A-linker key (bases represented in bold fonts), common for all sample primers, (ii) a 10-mer barcode unique to each sample primer followed by a common 3-mer barcode adaptor (all marked as stars), and finally (iii) the relevant domain-specific universal forward primer in its 5′ to 3′ direction (underlined bases). Reverse primers, in their turn, had (i) a common trP1 adapter (bases represented in italics), followed by (ii) the relevant domain-specific universal reverse primer in its 5′ to 3′ direction (underlined bases).

V3 regions of all bacterial 16S rRNA genes present in a metagenome were amplified using the forward primer 5′-CCA TCT CAT CCC TGC GTG TCT CCG ACT CAG *** *** *** *** *CC TAC GGG AGG CAG CAG-3′ (where the underlined portion represents the universal primer 341 f) and the reverse primer 5′-*CCT CTC TAT GGG CAG TCG GTG AT*A TTA CCG CGG CTG CTG G-3′ (where the underlined portion represents the universal primer 515r). V3 regions of all archaeal 16S rRNA genes present in a metagenome were amplified using the forward primer 5′-CCA TCT CAT CCC TGC GTG TCT CCG ACT CAG *** *** *** *AA TTG GAN TCA ACG CCG G-3′ (where the underlined portion represents the universal primer 344 f) and the reverse primer 5′-*CCT CTC TAT GGG CAG TCG GTG A*TC GRC GGC CAT GCA CCW C-3′ (where the underlined portion represents the universal primer 522r).

Each 50 µl PCR reaction contained 10 µl template (corresponding to ∼100 ng metagenomic DNA), 5 µl 10 × KOD DNA polymerase buffer, 5 µl dNTP (0.25 mM each), 2 µl MgCl_2_ (25 mM), 1.5 µl (3%) DMSO, 3 µl each of the forward and reverse primer (0.3 µM each), 19.5 µl dH_2_O and 1 µl KOD Hot Start DNA Polymerase enzyme (Novagen, Merck Biosciences, Darmstadt, Germany). PCR products were amplified for 30 cycles as follows: 95 °C for 20S, 65 °C for 30 s and 70 °C for 10 s. After amplification, all PCR products were electrophoresed on 2.5% w/v agarose gel, purified by size selection, and adjusted to final concentrations of 10 ng μl^−1^ using molecular grade water. PCR products from all the samples were pooled up at equal concentrations for subsequent Ion PGM sequencing.

Before Ion PGM sequencing, size distribution and DNA concentration in the pooled-up amplicon mixture was examined using a Bioanalyzer 2100 (Agilent Technologies, Santa Clara, California, USA). The mixed sample was adjusted to a final concentration of 26 pM and attached to the surface of Ion Sphere Particles (ISPs) using an Ion Onetouch 200 Template kit (Thermo Fisher Scientific, Waltham, Massachusetts, USA) according to the manufacturer’s instructions. Manual enrichment of the resulting ISPs resulted in >95% templated-ISPs, which were then sequenced on Ion 316 Chips using the Ion PGM (Ion Express Template 200 chemistry) for 500 flows that gives an expected average read length of >220 bp. Post sequencing, and before retrieval from the sequencing machine, all reads were filtered by the inbuilt PGM software to remove low-quality- and polyclonal-sequences; sequences matching the PGM 3′ adaptor were also automatically trimmed. Every V3 sequence dataset after this quality-filtration was exported as a fastq file for downstream analyses; at the same time all were deposited to the Sequence Read Archive (SRA) of the National Center for Biotechnology Information (NCBI) with individual run accession numbers (given in Supplementary Tables 3–8) under the BioProject accession number PRJNA309469.

### Clustering of V3 reads into OTUs

Every V3 sequence dataset retrieved from the Ion PGM was further filtered for quality value 20 and read-length threshold of 100 nucleotides. Selected reads were converted to fasta using Fastx_toolkit 0.0.13.2 (http://hannonlab.cshl.edu/fastx_toolkit/download.html). Subsequently preprocessing was done using the “preproc” module of ESPRIT^90^; this involved the screening of only such reads that had intact 16S rDNA universal primer sequence plus the barcode adaptor and the barcode. Adaptor sequences were clipped off every individual read, subsequent to which all such reads were removed from the dataset that contained unidentified bases or were identical to another read, or were sheer subsets of a longer read. After preprocessing, denoising of reads was carried out as follows: (i) pairwise-alignment of reads was carried out following Needleman algorithm using the module “pairwise.seqs” available in MOTHUR^91^; (ii) the alignment matrix created in this way was utilized for Single Linkage Preclustering^92^ using a script^93^ provided by the VAMPS (Visualization and Analysis of Microbial Population Structure project) integrated collection of tools http://vamps.mbl.edu/overview.php. Pairwise distance criterion of 0.02 (98% identity) was used to select reads in preclustering, which were then saved in fasta format. The purpose of this preclustering was to preempt the possibility of diversity-overestimation emanating from the potential 2% sequencing error of the Ion PGM platform.

Hierarchical clustering of reads was carried out using various modules available in ESPRIT^90^. During this process, reads were again aligned pairwise following Needleman algorithm, clusters or operational taxonomic units (OTUs) created with a pairwise distance criterion of 0.03 (97% identity), and finally the total number of OTUs or species-level entities determined. OTUs formed in this way were further used to draw statistical inferences. OTUs were filtered using a Perl script to remove all the singletons. Singletons were removed from*.Cluster as well as*.Cluster_List to make new_Cluster & new_Cluster_List files. The new Cluster files were run in the statistical module of ESPRIT to get ACE and Rarefaction analyses. The rarefaction information was further used in R package^94^ to plot for the number of reads taking part in OTU-formation against the number of OTUs formed. The clusters (minus singletons) were used together with the fasta and frequency files created during the clustering operations to create consensus sequences, which in turn were taxonomically classified with the help of the “RDP Classifier” located at http://rdp.cme.msu.edu/classifier/classifier.jsp.

### Species-richness estimation for sulfate-reducing bacteria

To estimate the species-richness (or total OTU-count) of sulfate-reducing bacteria within a given sediment-community, the total number of OTUs affiliated to such taxa were enumerated (from the RDP-classified OTU-set of that community), every member of which are known to reduce sulfate dissimilatorily. These included the genera *Desulfacinum*, *Desulfobacca*, *Desulfobaculum*, *Desulfocurvus*, *Desulfoglaeba*, *Desulfomonas*, *Desulfomonile*, *Desulforhabdus*, *Desulfosoma*, *Desulfovibrio*, *Desulfovirga*, *Desulfurella*, *Desulfuromonas*, *Desulfuromusa* and *Thermodesulforhabdus*; plus the entire families *Desulfarculaceae*, *Desulfobacteraceae*, *Desulfobulbaceae*, *Desulfohalobiaceae*, *Desulfomicrobiaceae* and *Desulfonatronaceae* of *Deltaproteobacteria*^95^. Notably, the OTU-set of none of the explored sediment-communities encompassed any consensus sequence ascribable to the SRB genera of the other bacterial phyla *Chrysiogenetes*, *Firmicutes*, *Nitrospiraea*, *Synergistetes* or *Thermodesulfobacteria*.

### Mathematical analysis of SRB diversity distribution

Probability density functions of the trends of variation observed in the distribution of SRB-OTUs along each sediment core were determined via the following steps. (I) First, the SRB-OTU-count profile of the core was segmented into apparent zones of fluctuation along the sediment-surface to core-bottom trajectory. This was done by referring to the relevant histogram within column I of Fig. 3 (each of these histograms was derived based on the normalized values of the SRB-OTU-counts for the various sediment-datapoints of the core; normalization of the SRB-OTU-count for a given datapoint was carried out by taking its ratio with the highest SRB-OTU-count obtained within that core). (II) In the second step, independent prediction of an approximate probability density function was done for every fluctuation-zone identified. (III) Finally, optimization of every probability density function was done with reference to the histogram through minimization of the χ^2^ value. Levenberg-Marquardt algorithm was used for χ^2^ minimization^96,97^ and the work was done using the software OriginPro 9 in a computer having an Intel(R) Core(TM) i5-2450 64-bit CPU and 8 GB RAM. In order to ensure that the theoretical density functions were best fitted, up to 4000 independent iterations were performed on every dataset. For each calculation, tolerance level of the reduced χ^2^ value was considered at 10^−9^.

### Data availability

All data supporting the findings of this paper are available within the Article and Supplementary Information files. DNA sequence data pertaining to the microbial diversity study are available from the Sequence Read Archive (SRA) of the National Center for Biotechnology Information (NCBI), a part of the US National Library of Medicine (NLM), a branch of the National Institutes of Health, USA: relevant accession numbers are all listed in Supplementary Tables 3–8 within the Supplementary Information files.

## Electronic supplementary material

Supplementary information

Dataset1

dataset 2
