# Supplementary material for: Enhanced carbon-sulfur cycling in the sediments of Arabian Sea oxygen minimum zone center
Source: Sci Rep. 2018 Jun 6;8:8665. doi: 10.1038/s41598-018-27002-2 (PMC5989202; doi:10.1038/s41598-018-27002-2)
Supplement: Supplementary file 1 — Supplementary information [file 41598_2018_27002_MOESM1_ESM.doc]

**Supplementary Information**

**Heightened carbon-sulfur cycling in sediments of Arabian Sea oxygen minimum zone center**

Svetlana Fernandes ^1^, Aninda Mazumdar^1*^, Sabyasachi Bhattacharya^2^, Aditya Peketi^1^, Tarunendu Mapder^3,#^, Rimi Roy^2^, Mary Ann Carvalho^1^, Chayan Roy^2^, P. Mahalakshmi^5^, Rheane Da Silva^1^, P. L. Srinivasa Rao ^6^, Suman Kumar Banik^3^, Wriddhiman Ghosh^2*^

^1^CSIR-National Institute of Oceanography, Dona Paula, Goa-403004, India

^2^Department of Microbiology, Bose Institute, Kolkata-700054, West Bengal, India

^3^Department of Chemistry, Bose Institute, 93/1 APC Road, Kolkata-700009, India

^4^Indian Institute of Engineering Science and Technology, Shibpur, Howrah-711103, West Bengal, India

^5^Prima Dona Homes, Dona Paula, Goa-403004, India

^6^Gujarat Energy Research and Management Institute, Gujarat- 382421, India

*Corresponding author: maninda@nio.org, wriman@jcbose.ac.in

**Contents**

**Supplementary Figure 1 and 2**

**Supplementary Figure 1.** Scatter plot of (TOC/TN)_molar_ versus δ^13^C_TOC_ of the studied sediment cores (SSK42/1-8).

**Supplementary Figure 2.** Pore-water ammonium concentrations and sediment TOC contents down the sediment-depths of SSK42/1-8.

**Supplementary Tables 1 - 10**

**Supplementary Table 1.** Latitude, longitude, core-length and water-depth of the gravity cores (SSK42/1-8) collected under this study.

**Supplementary Table 2.** Concentrations and isotope ratios of sediment organic matter and various porefluid components in the cores SSK42/1-8. Since this Table is longer than one page it has been provided as an Excel file.

**Supplementary Tables 3-8.** Sediment-depths of SSK42/1, 3, 5, 6, 7 and 8, respectively, which were explored for their microbiology; the basic statistics of OTU analysis of the respective samples are also given.

**Supplementary Table 9.** Number of OTUs affiliated to all *Bacteria* and sulfate-reducing bacteria at various sediment-depths of the coring-stations studied for microbiology.

**Supplementary Table 10-15.** Detection of genera encompassing fermentative, exoelectrogenic, and obligately/facultatively anaerobic sulfur-chemolithotrophic, bacteria down the sediment depth of SSK42/1, 3, 5, 6, 7 and 8, respectively.

.

**Supplementary Figure 1. :** Scatter plot of (TOC/TN)_molar_ versus δ^13^C_TOC_ of the studied sediment cores (SSK42/1-8). Dotted lines show approximate end member δ^13^C values of terrestrial C3, C4 vegetation and marine phytoplankton. RF = Redfield Ratio.

**Supplementary Figure 2.** Pore-water ammonium concentrations (a) and sediment TOC contents (b) down the sediment-depths of SSK42/1-8.

**Supplementary Tables 1 - 10**

**Supplementary Table 1.** Latitude, longitude, core-length and water-depth of the gravity cores (SSK42/1-8) collected under this study.

| **Station** | **Latitude** | **Longitude** | **Core length (in m)** | **Water depth**  **(in mbsl)** |
| --- | --- | --- | --- | --- |
| **SSK42/1** | 16ᵒ 49.64’ N | 71ᵒ 47.86’ E | 1.25 | 1275 |
| **SSK42/2** | 16ᵒ 49.65’ N | 71ᵒ 50.90’ E | 2.55 | 1089 |
| **SSK42/3** | 16ᵒ 49.93’ N | 71ᵒ 55.42’ E | 2.96 | 753 |
| **SSK42/4** | 16ᵒ 49.94’ N | 71ᵒ 56.84’ E | 2.2 | 688 |
| **SSK42/5** | 16ᵒ 49.88’ N | 71ᵒ 58.55’ E | 2.9 | 580 |
| **SSK42/6** | 16ᵒ 50.03’ N | 71ᵒ 59.50’ E | 2.95 | 530 |
| **SSK42/7** | 16ᵒ 50.03’ N | 72ᵒ 00.13’ E | 2.4 | 396 |
| **SSK42/8** | 17ᵒ 02.98’ N | 71ᵒ 56.41’ E | 2.8 | 225 |

**Supplementary Table 3.** Sediment-depths of SSK42/1 that were explored for their microbiology; the basic statistics of OTU analysis of the respective samples are also given.

| **Sediment-depths** | **BioSample accession numbers** | **Statistics of16S rRNA gene V3 region-based OTU clustering** | | | |
| --- | --- | --- | --- | --- | --- |
|  |  | **Run accession number** | **Total OTUs formed** | **Singletons** | **OTU count**  **(minus singletons)** |
| 8 cm | SAMN04442146 | SRR3501070 | 7,110 | 5,598 | 1,512 |
| 10 cm | SAMN04442147 | SRR3501075 | 5,481 | 4,299 | 1,182 |
| 21 cm | SAMN04442148 | SRR3501079 | 4,970 | 3,853 | 1,117 |
| 32 cm | SAMN04442149 | SRR3501087 | 5,834 | 4,485 | 1,349 |
| 40 cm | SAMN04442150 | SRR3501090 | 5,784 | 4,492 | 1,292 |
| 63 cm | SAMN04442151 | SRR3501092 | 3,253 | 2,501 | 752 |
| 84 cm | SAMN04442152 | SRR3501094 | 2,038 | 1,542 | 496 |
| 102 cm | SAMN04442153 | SRR3501095 | 4,181 | 3,216 | 965 |
| 125 cm | SAMN04442154 | SRR3501099 | 7,267 | 5,608 | 1,659 |

**Supplementary Table 4.** Sediment-depths of SSK42/3 that were explored for their microbiology; the basic statistics of OTU analysis of the respective samples are also given.

| **Sediment-depths** | **BioSample accession numbers** | **Statistics of16S rRNA gene V3 region-based OTU clustering** | | | |
| --- | --- | --- | --- | --- | --- |
|  |  | **Run accession number** | **Total OTUs formed** | **Singletons** | **OTU count (minus singletons)** |
| 8 cm | SAMN04442155 | SRR3823461 | 23,895 | 20,521 | 3,374 |
| 20 cm | SAMN04442156 | SRR3823462 | 9,706 | 8,439 | 1,267 |
| 35 cm | SAMN04442157 | SRR3823463 | 7,397 | 6,402 | 995 |
| 50 cm | SAMN04442158 | SRR3823464 | 10,230 | 8,921 | 1,309 |
| 65 cm | SAMN04442159 | SRR3823478 | 4,779 | 4,291 | 488 |
| 80 cm | SAMN04442160 | SRR3823506 | 6,169 | 5,496 | 673 |
| 95 cm | SAMN04442161 | SRR3823536 | 7,758 | 6,915 | 843 |
| 110 cm | SAMN04442162 | SRR3823537 | 8,838 | 7,864 | 974 |
| 125 cm | SAMN04442163 | SRR3823540 | 6,148 | 5,605 | 543 |
| 140 cm | SAMN04442164 | SRR3823543 | 5,969 | 5,419 | 550 |
| 150 cm | SAMN04442165 | SRR3823547 | 8,453 | 7,867 | 586 |
| 165 cm | SAMN04442166 | SRR3823548 | 5,475 | 5,050 | 425 |
| 180 cm | SAMN04442167 | SRR3823549 | 8,562 | 8,019 | 543 |
| 195 cm | SAMN04442168 | SRR3823550 | 5,716 | 5,322 | 394 |
| 210 cm | SAMN04442169 | SRR3823551 | 7,461 | 6,866 | 595 |
| 225 cm | SAMN04442170 | SRR3823552 | 6,359 | 5,911 | 448 |
| 240cm | SAMN04442171 | SRR3823553 | 8,825 | 8,116 | 709 |
| 255 cm | SAMN04442172 | SRR3823554 | 5,118 | 4,786 | 332 |
| 270 cm | SAMN04442173 | SRR3823555 | 6,886 | 6,489 | 397 |
| 285 cm | SAMN04442174 | SRR3823556 | 5,722 | 5,217 | 505 |

**Supplementary Table 5.** Sediment-depths of SSK42/5 that were explored for their microbiology; the basic statistics of OTU analysis of the respective samples are also given.

| **Sediment-depths** | **BioSample accession numbers** | **Statistics of16S rRNA gene V3 region-based OTU clustering** | | | |
| --- | --- | --- | --- | --- | --- |
|  |  | **Run accession number** | **Total OTUs formed** | **Singletons** | **OTU count (minus singletons)** |
| 0 cm | SAMN04442175 | SRR3646166 | 15,093 | 11,817 | 3,276 |
| 15 cm | SAMN04442176 | SRR3646167 | 7,217 | 5,750 | 1,467 |
| 45 cm | SAMN04442177 | SRR3646168 | 9,990 | 8,125 | 1,865 |
| 60 cm | SAMN04442178 | SRR3646174 | 11,160 | 8,758 | 2,402 |
| 90 cm | SAMN04442179 | SRR3646175 | 4,408 | 3,340 | 1,068 |
| 120 cm | SAMN04442180 | SRR3646176 | 10,553 | 8,298 | 2,255 |
| 140 cm | SAMN04442181 | SRR3646177 | 8,920 | 7,139 | 1,781 |
| 160 cm | SAMN04442182 | SRR3646178 | 3,302 | 2,573 | 729 |
| 190 cm | SAMN04442183 | SRR3646179 | 3,487 | 2,520 | 967 |
| 220 cm | SAMN04442184 | SRR3646180 | 9,087 | 7,419 | 1,668 |
| 260 cm | SAMN04442185 | SRR3646182 | 15,461 | 12,521 | 2,940 |
| 295 cm | SAMN04442186 | SRR3646183 | 14,423 | 11,875 | 2,548 |

**Supplementary Table 6.** Sediment-depths of SSK42/6 that were explored for their microbiology; the basic statistics of OTU analysis of the respective samples are also given.

| **Sediment-depths** | **BioSample accession numbers** | **Statistics of16S rRNA gene V3 region-based OTU clustering** | | | |
| --- | --- | --- | --- | --- | --- |
|  |  | **Run accession number** | **Total OTUs formed** | **Singletons** | **OTU count (minus singletons)** |
| 2 cm | SAMN04442187 | SRR3570042 | 7,980 | 5,835 | 2,145 |
| 15 cm | SAMN04442188 | SRR3576762 | 6,326 | 4,605 | 1,721 |
| 30 cm | SAMN04442189 | SRR3576763 | 6,088 | 4,423 | 1,665 |
| 45 cm | SAMN04442190 | SRR3576765 | 8,457 | 6,189 | 2,268 |
| 60 cm | SAMN04442191 | SRR3576766 | 2,804 | 2,094 | 710 |
| 75 cm | SAMN04442192 | SRR3576769 | 4,958 | 3,636 | 1,322 |
| 90 cm | SAMN04442193 | SRR3576774 | 2,422 | 1,756 | 666 |
| 105 cm | SAMN04442194 | SRR3576789 | 3,842 | 2,671 | 1,171 |
| 120 cm | SAMN04442195 | SRR3576794 | 3,352 | 2,305 | 1,047 |
| 135 cm | SAMN04442196 | SRR3576797 | 4,954 | 3,571 | 1,383 |
| 145 cm | SAMN04442197 | SRR3576799 | 4,756 | 3,668 | 1,088 |
| 160 cm | SAMN04442198 | SRR3576801 | 3,476 | 2,428 | 1,048 |
| 175 cm | SAMN04442199 | SRR3576803 | 4,261 | 3,084 | 1,177 |
| 190 cm | SAMN04442200 | SRR3577051 | 2,886 | 2,151 | 735 |
| 205 cm | SAMN04442201 | SRR3577340 | 2,664 | 2,017 | 647 |
| 220 cm | SAMN04442202 | SRR3577053 | 2,648 | 1,931 | 717 |
| 235 cm | SAMN04442203 | SRR3577056 | 3,092 | 2,321 | 771 |
| 250 cm | SAMN04442204 | SRR3577058 | 3,909 | 2,926 | 983 |
| 265 cm | SAMN04442205 | SRR3577060 | 4,337 | 3,236 | 1,101 |
| 270 cm | SAMN04442206 | SRR3577063 | 3,736 | 2,743 | 993 |
| 275cm | SAMN04442207 | SRR3884560 | 4,372 | 3,191 | 1,181 |

**Supplementary Table 7.** Sediment-depths of SSK42/7 that were explored for their microbiology; the basic statistics of OTU analysis of the respective samples are also given.

| **Sediment-depths** | **BioSample accession numbers** | **Statistics of16S rRNA gene V3 region-based OTU clustering** | | | |
| --- | --- | --- | --- | --- | --- |
|  |  | **Run accession number** | **Total OTUs formed** | **Singletons** | **OTU count (minus singletons)** |
| 15 cm | SAMN04442208 | SRR3823635 | 2,409 | 1,769 | 640 |
| 30 cm | SAMN04442209 | SRR3823636 | 2,422 | 1,868 | 554 |
| 45 cm | SAMN04442210 | SRR3823638 | 2,502 | 1,897 | 605 |
| 60 cm | SAMN04442211 | SRR3823639 | 2,491 | 1,872 | 619 |
| 70 cm | SAMN04442212 | SRR3823642 | 7,608 | 5,675 | 1,933 |
| 90 cm | SAMN04442213 | SRR3823643 | 5,945 | 4,462 | 1,483 |
| 105 cm | SAMN04442214 | SRR3823644 | 3,776 | 2,747 | 1,029 |
| 120 cm | SAMN04442215 | SRR3823646 | 3,619 | 2,740 | 879 |
| 145 cm | SAMN04442216 | SRR3823647 | 2,407 | 1,755 | 652 |
| 150 cm | SAMN04442217 | SRR3823648 | 3,014 | 2,208 | 806 |
| 165 cm | SAMN04442218 | SRR3823649 | 3,974 | 2,795 | 1,179 |
| 180 cm | SAMN04442219 | SRR3823650 | 3,558 | 2,478 | 1,080 |
| 210 cm | SAMN04442220 | SRR3823651 | 6,248 | 4,759 | 1,489 |
| 240 cm | SAMN04442221 | SRR3823654 | 3,263 | 2,514 | 749 |

**Supplementary Table 8.** Sediment-depths of SSK42/8 that were explored for their microbiology; the basic statistics of OTU analysis of the respective samples are also given.

| **Sediment-depths** | **BioSample accession numbers** | **Statistics of16S rRNA gene V3 region-based OTU clustering** | | | |
| --- | --- | --- | --- | --- | --- |
|  |  | **Run accession number** | **Total OTUs formed** | **Singletons** | **OTU count**  **(minus singletons)** |
| 2 cm | SAMN04442222 | SRR3865324 | 3,556 | 2,733 | 823 |
| 15 cm | SAMN04442223 | SRR3865686 | 4,147 | 3,118 | 1,029 |
| 45 cm | SAMN04442224 | SRR3868263 | 3,066 | 2,238 | 828 |
| 75 cm | SAMN04442225 | SRR3868265 | 2,156 | 1,611 | 545 |
| 105 cm | SAMN04442226 | SRR3868267 | 3,630 | 2,500 | 1,130 |
| 135 cm | SAMN04442227 | SRR3868268 | 4,759 | 3,509 | 1,250 |
| 150 cm | SAMN04442228 | SRR3868269 | 2,326 | 1,719 | 607 |
| 180 cm | SAMN04442229 | SRR3868271 | 2,875 | 2,075 | 800 |
| 210 cm | SAMN04442230 | SRR3868272 | 3696 | 2,627 | 1,069 |
| 240 cm | SAMN04442231 | SRR3868274 | 3,011 | 2,283 | 728 |
| 270 cm | SAMN04442232 | SRR3868276 | 2,547 | 1,921 | 626 |

**Supplementary Table 9.** Number of OTUs affiliated to all *Bacteria* and sulfate-reducing bacteria that were identified at the various sediment-depths (in cmbsf) of the coring-stations studied for microbiology.

| **SSK42/1** | | | | | | | | | | | | | | | | | | | | | |
| --- | --- | --- | --- | --- | --- | --- | --- | --- | --- | --- | --- | --- | --- | --- | --- | --- | --- | --- | --- | --- | --- |
| **Sediment-depth** | **8** | **10** | **21** | **32** | **40** | **63** | **84** | **102** | **125** |  | | | | | | | | | | | |
| **All *Bacteria*** | 1512 | 1182 | 1117 | 1349 | 1292 | 752 | 496 | 965 | 1659 |
| **SRB** | 105 | 62 | 29 | 24 | 27 | 3 | 0 | 48 | 25 |
| **SSK42/3** | | | | | | | | | | | | | | | | | | | | | |
| **Sediment-depth** | **8** | **20** | **35** | **50** | **65** | **80** | **95** | **110** | **125** | **140** | **150** | **165** | **180** | **195** | **210** | **225** | **240** | **255** | **270** | **285** |  |
| **All *Bacteria*** | 3374 | 1267 | 995 | 1309 | 488 | 673 | 843 | 974 | 543 | 550 | 586 | 425 | 543 | 394 | 595 | 448 | 709 | 332 | 397 | 505 |
| **SRB** | 240 | 57 | 44 | 79 | 10 | 26 | 24 | 56 | 30 | 8 | 13 | 21 | 6 | 11 | 22 | 14 | 16 | 19 | 16 | 31 |
| **SSK42/5** | | | | | | | | | | | | | | | | | | | | | |
| **Sediment-depth** | **0** | **15** | **45** | **60** | **90** | **120** | **140** | **160** | **190** | **220** | **260** | **295** |  | | | | | | | | |
| **All *Bacteria*** | 3276 | 1467 | 1865 | 2402 | 1068 | 2255 | 1781 | 729 | 967 | 1668 | 2940 | 2548 |
| **SRB** | 351 | 38 | 37 | 147 | 30 | 52 | 29 | 6 | 14 | 10 | 68 | 11 |
| **SSK42/6** | | | | | | | | | | | | | | | | | | | | | |
| **Sediment-depth** | **2** | **15** | **30** | **45** | **60** | **75** | **90** | **105** | **120** | **135** | **145** | **160** | **175** | **190** | **205** | **220** | **235** | **250** | **265** | **270** | **275** |
| **All *Bacteria*** | 2145 | 1721 | 1665 | 2268 | 710 | 1322 | 666 | 1171 | 1047 | 1383 | 965 | 1048 | 1177 | 735 | 647 | 717 | 771 | 983 | 1101 | 993 | 1181 |
| **SRB** | 70 | 32 | 37 | 33 | 7 | 12 | 10 | 14 | 5 | 9 | 48 | 11 | 22 | 13 | 9 | 6 | 15 | 27 | 14 | 13 | 26 |
| **SSK42/7** | | | | | | | | | | | | | | | | | | | | | |
| **Sediment-depth** | **15** | **30** | **45** | **60** | **70** | **90** | **105** | **120** | **145** | **150** | **165** | **180** | **210** | **240** |  | | | | | | |
| **All *Bacteria*** | 640 | 554 | 605 | 619 | 1933 | 1483 | 1029 | 879 | 652 | 806 | 1179 | 1080 | 1489 | 749 |
| **SRB** | 4 | 25 | 7 | 11 | 6 | 10 | 7 | 7 | 7 | 1 | 2 | 6 | 21 | 20 |
| **SSK42/8** | | | | | | | | | | | | | | | | | | | | | |
| **Sediment-depth** | **2** | **15** | **45** | **75** | **105** | **135** | **150** | **180** | **210** | **240** | **270** |  | | | | | | | | | |
| **All *Bacteria*** | 823 | 1029 | 828 | 545 | 1130 | 1250 | 607 | 800 | 1069 | 728 | 626 |
| **SRB** | 41 | 7 | 10 | 7 | 2 | 5 | 3 | 0 | 6 | 2 | 3 |

**Supplementary Table 10.** Detection of genera encompassing fermentative, exoelectrogenic, and obligately/facultatively anaerobic sulfur-chemolithotrophic, bacteria down the sediment depth of SSK42/1.

| **Genera identified** | **8**  **cm** | **10**  **cm** | **21**  **cm** | **32**  **cm** | **40**  **cm** | **63**  **cm** | **84**  **cm** | **102**  **cm** | **125**  **cm** |
| --- | --- | --- | --- | --- | --- | --- | --- | --- | --- |
| **Genera of homo- as well as hetero-fermentative bacteria** | | | | | | | | | |
| *Aliivibrio* | - | + | - | - | - | - | - | - | - |
| *Bacillus* | - | - | - | - | - | - | - | - | + |
| *Brachybacterium* | - | - | - | + | - | - | - | - | - |
| *Colwellia* | + | + | + | + | - | - | - | + | + |
| *Corynebacterium* | - | - | - | - | - | - | - | - | + |
| *Escherichia* | + | + | - | - | - | - | - | - | + |
| *Halomonas* | - | - | + | - | - | + | - | - | + |
| *Marinobacter* | - | + | + | - | - | - | - | - | + |
| *Pseudomonas* | - | - | + | - | - | + | - | - | + |
| *Staphylococcus* | - | + | - | - | + | - | - | - | + |
| **Genera of hydrogen-producing, exoelectrogenic bacteria** | | | | | | | | | |
| *Shewanella* | + | + | - | - | + | - | + | - | + |
| **Genera of obligately/facultatively anaerobic sulfur-chemolithotrophic bacteria** | | | | | | | | | |
| *Arcobacter* | + | - | - | - | - | - | - | + | + |
| *Paracoccus* | + | - | - | - | - | - | - | - | - |
| *Sulfurimonas* | - | - | - | - | - | - | - | - | + |
| *Thiohalomonas* | - | - | + | - | - | - | - | - | - |
| *Thiohalophilus* | - | - | - | + | - | - | - | - | - |

| **Genera identified** | 08 cm | 20 cm | 35 cm | 50 cm | 65 cm | 80 cm | 95 cm | 110 cm | 125 cm | 140 cm | 150 cm | 165 cm | 180 cm | 195 cm | 210 cm | 225 cm | 240 cm | 255 cm | 270 cm | 285 cm |
| --- | --- | --- | --- | --- | --- | --- | --- | --- | --- | --- | --- | --- | --- | --- | --- | --- | --- | --- | --- | --- |
| **Genera of homo- as well as hetero-fermentative bacteria** | | | | | | | | | | | | | | | | | | | | |
| *Bacillus* | - | - | - | + | + | - | - | - | - | + | - | - | - | - | - | + | + | - | + | - |
| *Bifidobacterium* | - | - | - | - | - | - | - | - | - | - | - | - | + | - | - | - | - | - | - | - |
| *Clostridium* | + | + | - | + | - | - | + | - | - | - | - | - | - | - | + | - | - | - | - | - |
| *Colwellia* | + | + | + | + | + | - | + | + | + | + | + | + | + | + | + | + | + | - | + | - |
| *Corynebacterium* | - | - | - | - | - | - | + | - | - | - | - | - | + | + | - | - | + | - | - | - |
| *Escherichia* | + | + | + | + | - | - | + | - | - | - | - | + | + | + | - | + | + | - | + | + |
| *Halomonas* | + | + | + | + | - | + | + | - | + | - | + | - | + | - | - | - | + | - | - | - |
| *Kocuria* | + | - | - | - | - | - | - | - | - | - | - | - | - | - | - | - | - | - | - | - |
| *Marinobacter* | + | - | - | - | - | - | - | - | - | - | + | - | + | + | - | - | - | - | + | - |
| *Microbacterium* | - | - | - | - | - | - | - | - | - | - | - | - | - | - | - | - | - | - | - | + |
| *Propionibacterium* | + | + | + | - | - | - | + | + | + | + | + | - | + | - | + | - | + | + | + | + |
| *Pseudomonas* | + | + | + | + | + | + | + | + | + | + | + | + | + | + | + | + | + | + | + | + |
| *Pseudovibrio* | - | - | - | - | - | - | - | - | - | - | - | - | - | - | + | + | - | + | - | - |
| *Serratia* | - | - | - | - | - | - | - | - | - | - | - | - | - | - | - | - | - | - | + | - |
| *Staphylococcus* | + | - | + | + | - | - | + | + | - | - | + | + | + | + | + | + | + | - | + | + |
| *Veillonella* | - | + | - | - | - | - | - | - | - | - | - | - | - | - | - | - | - | - | - | - |
| *Vibrio* | + | + | + | + | + | - | + | + | + | + | - | + | - | - | - | - | - | - | - | - |
| **Genera of hydrogen-producing, exoelectrogenic bacteria** | | | | | | | | | | | | | | | | | | | | |
| *Desulfovibrio* | + | + | + | + | - | + | - | + | + | - | - | + | - | - | - | - | - | + | - | - |
| *Shewanella* | + | - | - | - | + | - | + | - | - | + | - | + | - | + | - | - | - | - | - | - |
| **Genera of obligately/facultatively anaerobic sulfur-chemolithotrophic bacteria** | | | | | | | | | | | | | | | | | | | | |
| *Arcobacter* | + | + | + | + | + | + | - | + | - | - | - | - | - | - | - | - | - | - | - | + |
| *Desulfobulbus* | + | - | - | - | - | - | - | + | - | - | - | - | - | - | - | - | - | - | - | - |
| *Desulfopila* | - | - | - | - | - | - | - | + | - | - | - | - | - | - | - | - | - | - | - | - |
| *Desulforhopalus* | + | - | - | - | - | - | - | - | - | - | - | + | - | - | - | - | - | - | - | - |
| *Desulfotalea* | - | - | - | - | - | - | - | - | + | - | - | - | - | + | - | - | - | - | - | - |
| *Paracoccus* | - | - | - | + | - | - | - | - | - | - | - | - | + | + | - | - | - | - | - | - |
| *Sulfurimonas* | - | - | + | - | - | - | - | - | - | - | + | + | + | - | + | + | - | - | - | - |

**Supplementary Table 11.** Detection of genera encompassing fermentative, exoelectrogenic, and obligately/facultatively anaerobic sulfur-chemolithotrophic, bacteria down the sediment depth of SSK42/3.

**Supplementary Table 12.** Detection of genera encompassing fermentative, exoelectrogenic, and obligately/facultatively anaerobic sulfur-chemolithotrophic, bacteria down the sediment depth of SSK42/5.

| **Genera identified** | 0  cm | 15  cm | 45  cm | 60  cm | 90  cm | 120  cm | 140  cm | 160  cm | 190  cm | 220  cm | 260  Cm | 295  cm |
| --- | --- | --- | --- | --- | --- | --- | --- | --- | --- | --- | --- | --- |
| **Genera of homo- as well as hetero-fermentative bacteria** | | | | | | | | | | | | |
| *Arthrobacter* | - | + | + | - | - | - | - | - | - | - | - | - |
| *Brachybacterium* | - | + | - | - | - | - | - | - | - | - | - | - |
| *Colwellia* | + | - | + | - | + | + | + | + | + | + | + | - |
| *Enterococcus* | - | - | - | - | - | - | + | - | - | - | - | - |
| *Escherichia* | + | - | - | + | + | - | + | - | + | - | - | + |
| *Halomonas* | - | + | - | + | - | - | + | + | + | - | + | - |
| *Kocuria* | - | - | - | - | - | - | + | - | - | - | - | - |
| *Marinobacter* | + | - | - | + | + | + | - | - | + | - | - | - |
| *Microbacterium* | - | + | - | - | - | - | - | - | - | - | - | - |
| *Micrococcus* | - | + | - | - | - | - | + | - | - | - | - | - |
| *Propionibacterium* | - | - | - | - | + | - | - | - | - | - | - | - |
| *Pseudomonas* | - | - | - | - | - | - | - | - | + | - | - | - |
| *Staphylococcus* | + | - | - | - | - | + | - | - | + | - | + | - |
| *Streptococcus* | - | - | - | - | - | - | - | - | + | - | - | - |
| *Veillonella* | - | - | - | - | - | - | - | - | + | - | - | - |
| **Genera of hydrogen-producing, exoelectrogenic bacteria** | | | | | | | | | | | | |
| *Desulfovibrio* | - | - | - | - | + | - | - | - | - | - | + | - |
| *Shewanella* | - | - | - | + | + | - | - | - | + | - | - | - |
| **Genera of obligately/facultatively anaerobic sulfur-chemolithotrophic bacteria** | | | | | | | | | | | | |
| *Arcobacter* | - | + | + | - | - | - | - | - | - | - | - | - |
| *Paracoccus* | - | + | - | - | - | - | - | - | - | - | + | - |
| *Sulfurimonas* | - | - | - | - | - | - | - | - | + | - | - | - |

**Supplementary Table 13.** Detection of genera encompassing fermentative, exoelectrogenic, and obligately/facultatively anaerobic sulfur-chemolithotrophic, bacteria down the sediment depth of SSK42/6.

| **Genera identified** | 2  cm | 15  cm | 30  cm | 45  cm | 60  cm | 75  cm | 90  cm | 105  cm | 120  cm | 135  cm | 145  cm | 160  cm | 175  cm | 190  cm | 205  cm | 220  cm | 235  cm | 250  cm | 265  cm | 270  cm | 275  cm |
| --- | --- | --- | --- | --- | --- | --- | --- | --- | --- | --- | --- | --- | --- | --- | --- | --- | --- | --- | --- | --- | --- |
| **Genera of homo- as well as hetero-fermentative bacteria** | | | | | | | | | | | | | | | | | | | | | |
| *Aeromonas* | + | - | - | - | + | - | - | - | - | - | - | - | - | - | - | + | - | - | - | - | - |
| *Aliivibrio* | - | - | - | - | + | - | - | - | - | - | - | - | - | - | - | - | - | - | - | - | - |
| *Bacillus* | - | + | + | - | - | - | - | - | + | - | - | + | + | + | - | + | + | + | - | + | + |
| *Brachybacterium* | - | - | - | - | - | - | - | - | - | - | - | - | - | - | - | - | - | - | - | - | + |
| *Clostridium* | + | - | - | - | - | - | - | - | - | - | - | - | + | - | - | - | - | + | + | - | + |
| *Colwellia* | + | + | + | - | - | - | - | - | - | - | - | - | - | - | - | - | - | - | + | - | - |
| *Corynebacterium* | - | - | - | - | - | - | - | - | - | - | - | - | - | + | - | - | + | + | - | - | - |
| *Enterococcus* | - | - | - | - | - | - | - | - | - | - | - | - | - | - | - | - | - | - | + | - | - |
| *Escherichia* | + | - | - | - | - | - | - | - | - | - | + | - | + | - | - | - | + | - | - | + | - |
| *Halomonas* | + | + | + | + | + | + | + | + | + | + | + | + | + | + | + | + | + | - | + | + | + |
| *Kocuria* | - | - | - | - | - | - | - | - | - | - | - | - | - | - | - | - | - | - | - | + | - |
| *Lactococcus* | - | - | - | - | - | - | - | - | - | - | - | - | - | - | - | - | - | - | - | - | + |
| *Marinobacter* | + | + | + | + | + | + | + | + | + | + | + | + | + | + | + | + | + | + | + | + | + |
| *Marinobacterium* | - | - | - | - | + | - | - | - | - | - | - | - | - | - | - | - | - | - | - | - | - |
| *Microbacterium* | - | - | + | - | - | - | - | - | - | - | - | - | - | - | - | - | - | - | - | - | + |
| *Propionibacterium* | + | + | + | - | - | - | - | - | - | - | - | + | - | - | - | + | - | + | - | - | - |
| *Pseudomonas* | + | - | - | + | - | + | + | - | + | + | + | - | - | - | + | - | + | - | + | + | - |
| *Staphylococcus* | + | + | + | + | - | + | - | + | + | + | + | + | + | + | + | + | + | + | + | + | + |
| *Streptococcus* | - | - | - | - | - | - | - | - | - | - | - | - | - | + | - | - | - | + | + | - | - |
| *Vibrio* | + | - | + | - | - | - | + | - | - | + | - | + | - | - | - | - | - | - | - | - | + |
| *Weissella* | - | - | - | - | - | - | - | - | - | - | - | - | - | - | - | - | - | + | - | - | - |
| **Genera of hydrogen-producing, exoelectrogenic bacteria** | | | | | | | | | | | | | | | | | | | | | |
| *Desulfovibrio* | - | - | - | + | - | - | - | - | - | - | - | - | - | - | - | - | - | - | - | - | - |
| *Geobacter* | - | - | - | - | - | - | - | - | - | - | - | - | - | - | - | - | - | + | - | - | - |
| *Shewanella* | + | + | - | + | + | + | + | + | + | - | + | + | + | - | - | - | - | - | + | - | - |
| **Genera of obligately/facultatively anaerobic sulfur-chemolithotrophic bacteria** | | | | | | | | | | | | | | | | | | | | | |
| *Arcobacter* | + | - | - | - | + | + | - | + | - | - | - | + | - | - | - | - | - | - | - | - | - |
| *Paracoccus* | - | - | - | - | - | - | - | - | - | - | - | - | + | - | - | - | - | + | - | - | - |
| *Sulfurimonas* | - | - | + | + | + | + | + | - | + | + | + | + | + | - | - | - | - | + | - | + | + |
| *Thiohalomonas* | - | - | - | - | - | - | - | - | + | - | - | - | - | - | - | - | - | - | - | - | - |

**Supplementary Table 14.** Detection of genera encompassing fermentative, exoelectrogenic, and obligately/facultatively anaerobic sulfur-chemolithotrophic, bacteria down the sediment depth of SSK42/7.

| **Genera identified** | 15 cm | 30 cm | 45 cm | 60 cm | 70 cm | 90 cm | 105 cm | 120 cm | 145 cm | 150 cm | 165 cm | 180 cm | 210 cm | 240 cm |
| --- | --- | --- | --- | --- | --- | --- | --- | --- | --- | --- | --- | --- | --- | --- |
| **Genera of homo- as well as hetero-fermentative bacteria** | | | | | | | | | | | | | | |
| *Bacillus* | - | - | - | - | - | - | - | - | - | + | - | - | - | - |
| *Bacteroides* | - | - | - | - | - | - | - | - | - | - | - | - | + | - |
| *Bifidobacterium* | - | - | - | - | - | - | - | - | - | - | - | - | + | - |
| *Brevibacterium* | - | - | - | - | - | - | - | - | - | - | - | - | + | - |
| *Carnobacterium* | - | - | - | - | - | - | - | - | - | - | - | - | + | - |
| *Cellvibrio* | - | - | - | - | - | - | - | - | - | - | - | - | + | - |
| *Clostridium* | - | - | - | - | - | - | + | + | - | - | - | - | + | - |
| *Colwellia* | - | - | + | + | + | - | - | + | - | + | - | - | - | - |
| *Corynebacterium* | - | - | - | - | + | + | - | - | - | - | - | + | + | - |
| *Enterococcus* | - | - | - | - | - | - | - | - | - | - | - | - | + | - |
| *Escherichia* | - | + | - | - | - | + | + | + | - | - | + | - | - | - |
| *Halomonas* | - | + | + | - | - | - | - | - | - | - | + | - | + | - |
| *Lactobacillus* | - | - | - | - | - | - | - | - | - | - | - | + | + | - |
| *Marinobacter* | + | + | + | - | + | - | - | + | + | + | + | - | + | - |
| *Propionibacterium* | + | - | - | - | - | - | - | - | - | - | - | - | - | - |
| *Pseudomonas* | - | + | + | + | + | - | + | + | + | - | + | + | + | - |
| *Staphylococcus* | + | + | + | + | + | + | + | + | + | + | + | + | + | + |
| *Streptococcus* | - | - | - | - | - | - | - | - | - | - | - | + | + | - |
| *Veillonella* | - | - | - | - | - | - | - | - | - | - | - | - | + | - |
| **Genera of hydrogen-producing, exoelectrogenic bacteria** | | | | | | | | | | | | | | |
| *Geobacter* | - | - | - | - | - | - | - | - | - | - | - | - | + | - |
| **Genera of obligately/facultatively anaerobic sulfur-chemolithotrophic bacteria** | | | | | | | | | | | | | | |
| *Arcobacter* | + | + | + | + | + | - | - | - | - | - | - | - | - | - |
| *Paracoccus* | - | - | - | - | - | - | - | - | - | - | + | - | + | - |
| *Sulfurimonas* | - | + | + | + | - | + | - | + | - | - | + | + | - | - |

**Supplementary Table 15.** Detection of genera encompassing fermentative, exoelectrogenic, and obligately/facultatively anaerobic sulfur-chemolithotrophic, bacteria down the sediment depth of SSK42/8.

| **Genera identified** | 02 cm | 15 cm | 45 cm | 75 cm | 105 cm | 135 cm | 150 cm | 180 cm | 210 cm | 240 cm | 270 cm |
| --- | --- | --- | --- | --- | --- | --- | --- | --- | --- | --- | --- |
| **Genera of homo- as well as hetero-fermentative bacteria** | | | | | | | | | | | |
| *Brachybacterium* | - | - | - | - | - | - | - | - | + | + | - |
| *Brevibacterium* | - | - | - | - | - | - | - | - | + | + | + |
| *Colwellia* | + | - | + | + | + | - | - | - | - | - | - |
| *Corynebacterium* | + | + | + | + | + | + | + | + | + | + | + |
| *Escherichia/Shigella* | - | + | - | + | + | + | + | - | + | + | + |
| *Halomonas* | + | + | + | - | + | + | + | - | + | - | + |
| *Marinobacter* | + | - | + | + | + | + | + | + | + | + | + |
| *Propionibacterium* | + | + | + | + | + | + | + | + | + | + | + |
| *Pseudomonas* | - | - | - | - | - | + | + | + | - | + | + |
| *Serratia* | - | + | - | - | - | - | - | - | - | - | + |
| *Staphylococcus* | + | + | - | + | + | + | + | + | + | + | + |
| *Streptococcus* | - | - | - | - | - | + | - | - | - | + | + |
| **Genera of obligately/facultatively anaerobic sulfur-chemolithotrophic bacteria** | | | | | | | | | | | |
| *Arcobacter* | + | - | - | - | - | - | - | - | - | - | - |
| *Paracoccus* | + | + | + | + | - | + | + | + | + | + | + |
| *Sulfurimonas* | + | + | + | - | - | - | - | - | - | - | - |
